# Locked Superior Dislocation of the Acromioclavicular Joint

**DOI:** 10.1155/2013/508219

**Published:** 2013-12-18

**Authors:** Salma Eltoum Elamin, Apurv Sinha, Mark Webb

**Affiliations:** Department of Trauma & Orthopaedics, Countess of Chester Hospital, Liverpool Road, Chester CH2 1UL, UK

## Abstract

Acromioclavicular (AC) joint injuries account for approximately 3–5% of shoulder girdle injuries (Rockwood et al., 1998). Depending on severity of injury and direction of displacement these are classified using Rockwood classification system for AC joint dislocation. We present an unusual case presenting with locked superior dislocation of the AC joint highlighting the presentation and subsequent successful surgical management of such case. To our knowledge this has not been reported previously in literature.

## 1. Introduction 

First described by Hippocrates, AC joint disruption usually results from a fall onto the point of the shoulder with subsequent disruption of the ligaments and muscular attachments stabilizing this joint [1].

**Figure 1:**
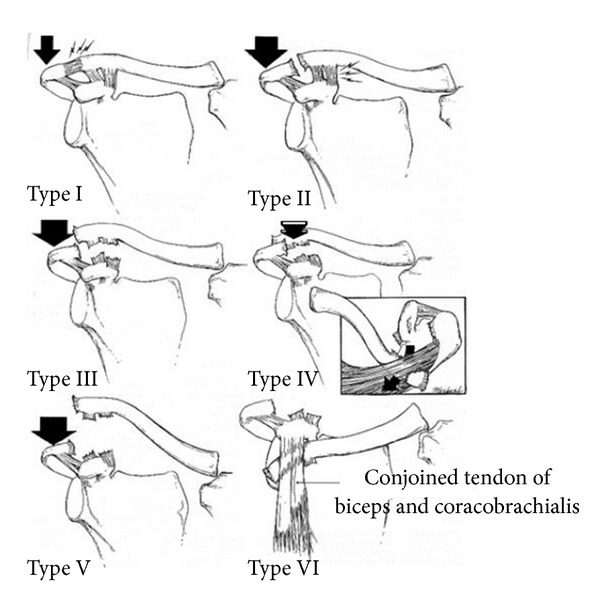
Rockwood's classification of the acromioclavicular joint injuries. (Reproduced from Bucholz RW, Heckman JD, Rockwood AJ, and Rockwood and Green's Fractures in Adults, vol. 1. Philadelphia: Lippincott Williams & Wilkins, 1991).

**Figure 2:**
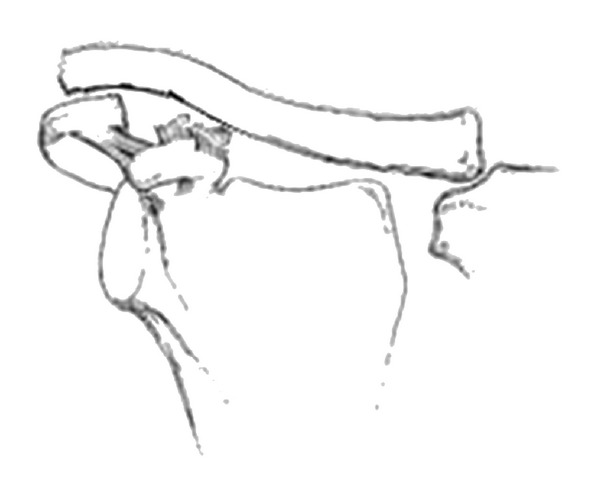
A schematic representation of the locked superior dislocation of the acromioclavicular joint.

Representing 3 to 5% of shoulder girdle injuries [2], it was first classified by Tossy & Allaman into three types depending on the degree of anatomical disruption which was reflected on the radiographic appearance of the joint [3, 4]. Rockwood further classified type 3 injuries into four types (3 to 6) (Figure 1). This classification is now routinely used to guide the management of AC joint injuries [2], where type 1 and 2 are managed conservatively and for type 4 to 6 operative reduction and stabilization is the recommended treatment option [5]. These various surgical techniques have been described in the literature including open and arthroscopic techniques [6]. We report a unique case of superior locked dislocation of the AC joint (Figure 2) whose severity was initially missed due to its unusual presentation but was subsequently managed operatively. To our knowledge such presentation of AC joint injury has not been reported in the past and the initial management was delayed due to failure to recognize the severity of injury.

## 2. Case Report 

**Figure 3:**
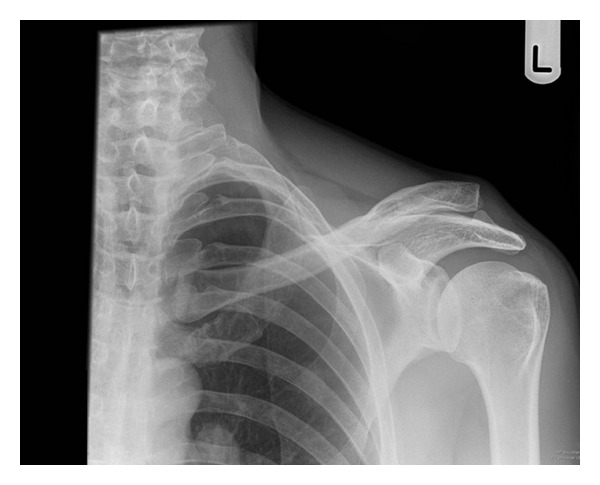
AP radiograph taken in emergency department at initial presentation.

**Figure 4:**
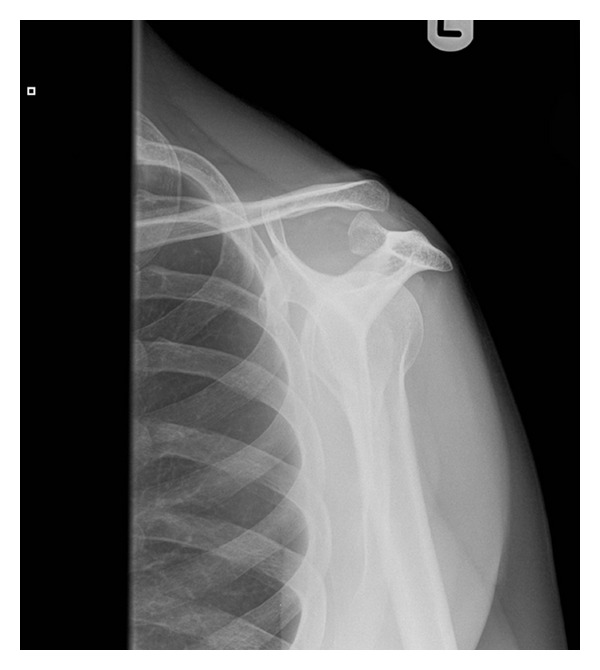
Y view radiograph taken in emergency department at initial presentation.

A 52-year-old right handed Caucasian male farmer fell off a galloping horse landing heavily on his left shoulder. He experienced immediate pain, swelling, and inability to move the shoulder. He originally presented to the emergency department on the same day and initial radiographic evaluation (AP and scapular Y views), Figures 3 and 4, showed dislocation of AC joint. He had no other injuries and had no active ongoing medical problems. He was subsequently provided with a broad arm sling and referred to orthopaedics where he was seen in fracture clinic five days after the initial injury. On presentation in fracture clinic he had swelling around the AC joint with significant pain and reduced range of movement in his left shoulder (30 deg. abduction, 40 deg. flexion, 30 deg. external rotation, and 10 deg. internal rotation). Neurovascular examination of the left upper extremity including the brachial plexus and axillary nerve was entirely normal. After assessment a diagnosis of type 2 ACJ dislocation was made and a trial of conservative management with a broad arm sling and early physiotherapy was organised. The patient was subsequently reviewed three weeks after the initial injury, when he complained of ongoing pain and loss of function with no improvement with physiotherapy.

**Figure 5:**
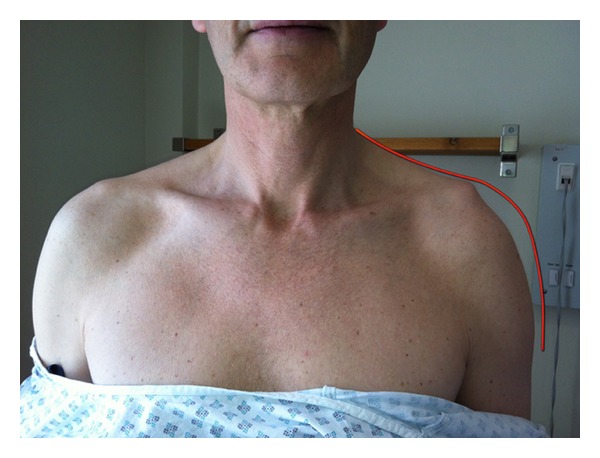
Image showing the asymmetry between the shoulders and red line illustrates the normal contour of the shoulder as drawn from the right shoulder.

**Figure 6:**
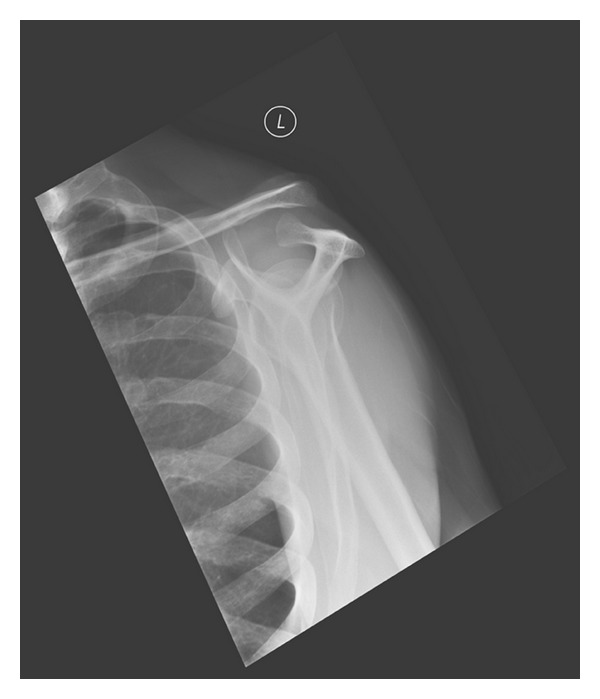
Y radiograph of the left shoulder taken 3 weeks after initial injury showing the dislocated acromioclavicular joint.

**Figure 7:**
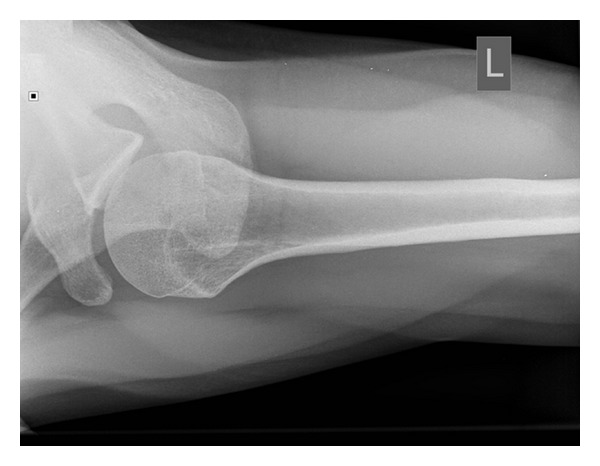
Axillary view radiograph taken 3 weeks after initial injury.

**Figure 8:**
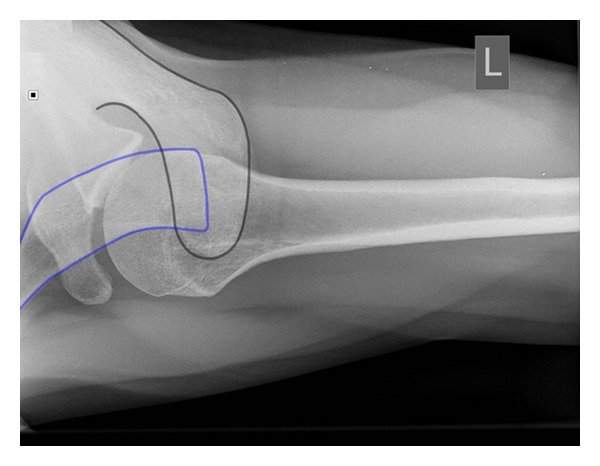
Axillary view radiograph taken 3 weeks after initial injury with the black blue lines illustrating the acromion and calvicle, respectively, confirming the direct superior position of the clavicle in relation to the acromion.

At this point the patient was referred to the senior author (MW) whose clinical evaluation confirmed a dislocated AC joint with secondary winging of the scapula. With improvement in swelling around shoulder a noticeable asymmetry of the AC joints was observed (Figure 5). Further radiographic imaging (Y view and axillary view), Figures 6, 7, and 8, confirmed this unusual form of AC joint dislocation with complete dislocation of the joint and the lateral end of the clavicle displaced and locked superiorly directly on top of the acromion. After consideration of patient level of activity and the irreducible locked dislocation, a decision was taken to proceed to open reduction of the joint and subsequent stabilization using the Nottingham Surgilig Ligament (Surgilig, Surgilig Craft, Redditech, UK). Surgery was performed five weeks after injury by the senior author (MW) under general anaesthetic with regional anaesthetic augmentation and intravenous antibiotic cover. The patient was positioned in beach chair position and a bra strap incision was utilised. A superiorly dislocated lateral end of the clavicle was identified. Both acromioclavicular and coracoclavicular ligaments were found to be disrupted but there was no periosteal stripping of lateral end of clavicle. It was not possible to reduce the clavicle so distal clavicular resection of approximately 1 cm was undertaken with subsequent reduction of the acromioclavicular joint. An 11 cm Surgilig ligament secured with a 3.5 × 36 mm fully threaded cortical screw with washer was used to stabilise the joint. Further augmentation and repair of superior acromioclavicular ligament was done using 1-0 PDS suture. Routine skin closure using subcuticular 4-0 monocryl and steri-stips was undertaken.

**Figure 9:**
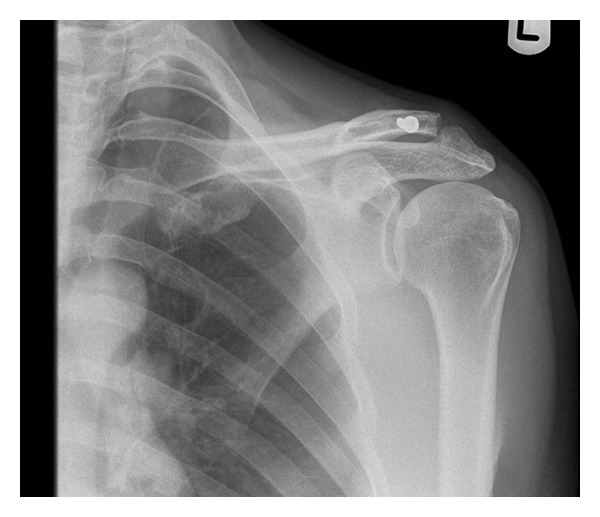
Postoperative AP radiograph showing the reduced joint.

**Figure 10:**
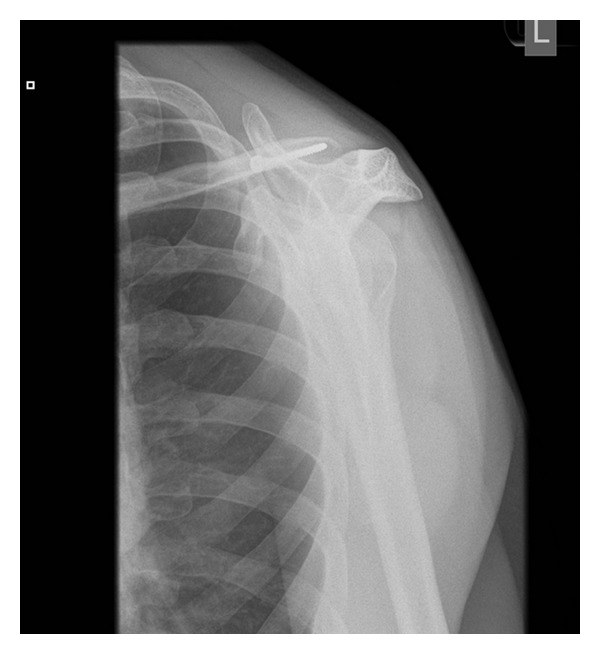
Postoperative Y view radiograph showing the reduced joint with screw in situ.

The patient was discharged the following day with a polysling to protect the repair. The initial exercise program included pendulum and single plane passive range of movement for the shoulder and the sling to be worn for 3 weeks. Active assisted exercises were commenced at 3 weeks postoperatively. Full activity was resumed at 8 weeks. At 2 years followup, the patient reported resumption of full activity with no pain. Clinical examination confirmed a full range of pain-free movement at the shoulder, with no residual scapular winging. Radiographs confirmed maintenance of the AC joint 93 stabilization, Figures 9 and 10. Outcome measures revealed an Oxford shoulder score of 43 (out of 60, i.e., satisfactory joint function) and University of California, Los Angeles (UCLA), shoulder score of 33 (excellent as per UCLA scoring being > 27).

## 3. Discussion 

The main classification system used for stratifying treatment options in AC joint separation is the Rockwood's classification system. Based on the sequential disruption of the stabilizing structures surrounding the joint 6 types were described from type 1 recognized as acromioclavicular ligament sprain, type 2 characterized by complete disruption of the acromioclavicular joint capsule and acromioclavicular ligaments resulting in instability of the joint in the horizontal plane with acromioclavicular subluxation (50% height of the clavicle). Type 3 being a complete separation of the joint with increase in the acromioclavicular distance resulting from complete disruption of the coracoclavicular ligament [1]. Distal clavicle is displaced posteriorly through the trapezius muscle in type 4 injuries. Type 5 shows severe stripping of the deltoid and trapezial fascia off the acromion as well as the lateral clavicle resulting in 2- to 3-fold increase of the coracoclavicular distance [2]. Type 6 being very rare usually results from severe hyperabducation and external rotation of the arm with subsequent subacromial or subcoracoid dislocation of the clavicle behind an intact conjoined tendon [2]. Fractures of the clavicle or coracoid have been associated with this type of injury [7]. Locked superior dislocation of the acromioclavicular joint has not been reported previously. The authors believe the mechanism of injury to be a force applied to the superior-lateral part of the acromion in an inferomedial direction, the applied force being sufficient to result in failure of both the acromioclavicular and the coracoclavicular ligament. The latter being the main restraint for medial displacement of the scapula, its loss leads to the displacement of the acromion medially. The lateral end of the clavicle being locked on top of the acromion maintains the dislocated position of the joint resulting in the gross deformity of the joint and painful restricted abduction of the shoulder. In our opinion, considering the observed pathology, this is unique presentation where the more inferiomedially directed force has been applied to the acromion resulting in medialisation of the acromion inferior to the clavicle as compared to a medially and anteriorly directed force in a Rockwood Type 4 injury. Clinically the involved shoulder is grossly deformed with an easily palpable lateral end of the clavicle with difficulty defining the borders of the acromion. We have not observed any neurovascular injury or concomitant injury of the sternoclavicular joint in the present case.

Surgical treatment was considered in view of the patient circumstances, degree of deformity, and the persistent dislocated position of the joint. Open reduction of the joint was attempted but was not possible without resection of the lateral end of the clavicle to free the acromion. The authors believe that the inability to reduce the joint is due to the nature of the locked dislocation of the joint and the delay in identifying the severity of injury and subsequent surgical procedure.

## 4. Conclusion 

We report this unusual case of superior locked dislocation of the AC joint and advocate including this as Type 7 of a modified Rockwood classification. This will hopefully increase awareness of such variant and prevent missed diagnosis and subsequently delayed or inappropriate management. We report good outcome following surgical management with open reduction and stabilisation of AC joint with coracoclavicular ligaments augmentation using the Nottingham Surgilig prosthetic ligament.
